# The Protein Data Bank in Europe (PDBe): bringing structure to biology

**DOI:** 10.1107/S090744491004117X

**Published:** 2011-03-18

**Authors:** Sameer Velankar, Gerard J. Kleywegt

**Affiliations:** a Protein Data Bank in Europe (PDBe), EMBL–EBI, Wellcome Trust Genome Campus, Hinxton, Cambridge CB10 1SD, England

**Keywords:** Protein Data Bank in Europe

## Abstract

The Protein Data Bank in Europe (PDBe) is the European partner in the Worldwide PDB and as such handles depositions of X-ray, NMR and EM data and structure models. PDBe also provides advanced bioinformatics services based on data from the PDB and related resources. Some of the challenges facing the PDB and its guardians are discussed, as well as some of the areas on which PDBe activities will focus in the future (advanced services, ligands, integration, validation and experimental data). Finally, some recent developments at PDBe are described.

## Abstract 2

Some future challenges for the PDB and its guardians are discussed and current and future activities in structural bioinformatics at the Protein Data Bank in Europe (PDBe) are described.

## 1. Introduction

The Protein Data Bank in Europe (PDBe; http://pdbe.org; Velankar *et al.*, 2010 ▶, 2011 ▶), previously known as the European Macromolecular Structure Database (MSD; Velankar *et al.*, 2005 ▶; Tagari *et al.*, 2006 ▶), is the European partner in the Worldwide Protein Data Bank (wwPDB; http://wwpdb.org; Berman *et al.*, 2007 ▶). Together with its wwPDB partners, RCSB (Kouranov *et al.*, 2006 ▶), PDBj (Standley *et al.*, 2008 ▶) and BMRB (Ulrich *et al.*, 2008 ▶), PDBe accepts depositions of experimentally determined biomacromolecular structures and the underlying experimental data. The deposition sites curate (*i.e.* annotate, enrich and validate) the structures and the data and make them available in the Protein Data Bank (PDB; Bernstein *et al.*, 1977 ▶; Berman, 2008 ▶). The PDB is the single worldwide archive of biomacromolecular structures. The master copy of the PDB resides on an ftp server hosted by RCSB, with mirrors at PDBe and PDBj. The weekly updates of the PDB are released by all of the wwPDB sites simultaneously at 0:00 UTC (Coordinated Universal Time) on Wednesdays. The archive is freely downloadable and is mirrored by many third-party sites. The wwPDB partners collaborate intensely on all matters related to ‘data-in’; that is, the deposition and annotation of structures and data and issues related to formats, standards, validation and the description of the ligands that occur in the PDB. However, as regards ‘data-out’ they each offer different and competing services, thus providing alternative ways of delivering and presenting the data in the PDB to the user community through their individual websites.

PDBe was established (initially as MSD) at the European Bioinformatics Institute (EMBL–EBI; http://www.ebi.ac.uk) in 1995. Under the leadership of Geoff Barton (1998–2001) and Kim Henrick (2001–2009), PDBe built up a reputation as a provider of advanced structural bioinformatics databases and services. PDBe also founded the Electron Microscopy Data Bank (EMDB; http://emdatabank.org; Tagari *et al.*, 2002 ▶; Henrick *et al.*, 2003 ▶) in 2002. However, like the PDB, EMDB is now an international collaborative effort involving PDBe, RCSB and the laboratory of Wah Chiu at Baylor College of Medicine (BCM) (Lawson *et al.*, 2011 ▶).

In this paper, we discuss some of the challenges facing the PDB and its guardians in the near future. Subsequently, we describe the areas in which PDBe has traditionally been strong or wants to become strong as well as some of our future plans in these areas. Finally, we discuss some recent developments at PDBe.

## 2. The future of the PDB

There is no doubt that the future of the PDB will involve growth: growth in the number of entries, growth in the size and complexity of entries and growth in the number of experimental techniques (including so-called hybrid methods) that are used to determine the structures of biologically relevant molecules, complexes and machines at anything from very low to atomic resolution. These developments will have profound consequences for PDBe, for wwPDB and for all producers and users of biomacromolecular structures. The wwPDB partners are currently developing a new common deposition and annotation tool that will enable them to handle a greater number of more complex and more diverse structures (and the underlying experimental data) produced by a number of different experimental methods. As structures have become larger and more complex, the limits of the original PDB format (Bernstein *et al.*, 1977 ▶) have been reached (and in some cases breached). For this reason, the wwPDB partners, in consultation with community stakeholders, are developing a new more versatile PDB format. Another important area in which the wwPDB partners have joined forces is the validation of structures and data (Kleywegt, 2009 ▶). wwPDB has established validation task forces (VTFs) for X-ray crystallography (Berman *et al.*, 2010 ▶), NMR spectroscopy and cryoelectron microscopy (EM). The recommendations of these task forces will be implemented as part of the deposition and annotation pipeline. The new system will include validation of small molecules bound to macromolecules. These small molecules form an increasingly important component of the structure data available in the archive, but validation of their structures and reliability has been problematic (Kleywegt, 2007 ▶). To remedy this problem, wwPDB has entered into a collaboration with the Cambridge Crystallographic Data Centre (CCDC; http://www.ccdc.cam.ac.uk/), giving the wwPDB partners access to CCDC tools and databases. In particular, *Mogul* (Bruno *et al.*, 2004 ▶), a program for validation of the geometry of small molecules, will become a crucial component of the wwPDB ligand-validation arsenal.

As the PDB approaches its 40th birthday in 2011, everybody involved with it faces an important question: should the PDB should remain a historic archive or not? The PDB has hitherto been an archive of the structural data as described in the primary literature. For instance, the coordinates available in the PDB for a structure determined in 1976 are those described in that year, even though model-building, refinement and validation methodology has advanced enormously since then. Even automatic approaches could produce improved models for the vast majority of crystal structures in the PDB today (Joosten *et al.*, 2009 ▶). The PDB is very much a provider-centric archive, which is great for crystallographers and historians but which severely limits the accessibility and usefulness of structural data to non-expert users. Shifts in user communities and user demands, as well as limits on the resources and funding sources available for the maintenance and development of the PDB, put pressure on the structural biology community as a whole to make its findings more accessible, and indeed relevant, to large groups of non-expert users (from medicinal chemists to geneticists and physicians). In our view, during its fifth decade the PDB should be transformed from a historical archive into a useful resource for biomedicine (and related fields such as agriculture). Non-expert users approach the structural archive very differently from structural biologists. They generally think in terms of genes, proteins, pathways, mutations or diseases, not in terms of PDB ID codes. Furthermore, non-experts have great difficulty in telling a good and reliable structure model of a certain molecule from a poor one. These observations have a number of implications for the way in which the structural archive needs to be organized and presented to such users.

(i) There is a need for new ways of searching for structural information. Whereas a structural biologist can usually locate a structure of a molecule or complex of interest using text-based or sequence searches, non-experts who think in terms of, for instance, pathways or diseases should be able to browse the structural knowledge base using concepts, terms and classifications that are relevant and familiar to them.
(ii) There is a need for new ways of handling structural information. Rather than a search or browse operation resulting in a list of hits (PDB entries that match a set of criteria), the non-expert user may want to take these hits and carry out certain tasks, ranging from visualization, superposition and structure-based sequence alignment to mapping of SNP data or comparison of binding sites. As the example of SNP mapping shows, structural data presented in isolation are of only limited use. Integration of multiple sources of bio­logical data and information will add significant value and will help non-expert users to answer complex questions that involve three-dimensional structure.
(iii) There is a need for best-practice structural models. A non-expert user will rarely be interested in the exact coordinates as they were deposited when a structure was published. Instead, they will assume that the model they download is the best possible interpretation of the experimental data obtained using state-of-the-art methods. Obviously, the historic data need to be available as well, but it is untenable in the long run to put the onus on the user when it comes to obtaining a best-practice model. Related to this is the need to provide information about the quality of models so that the most suitable model can be selected from amongst a number of alternatives, even by non-experts. This need will be addressed by the wwPDB VTFs.

Some of these requirements have implications for wwPDB as a whole, while others apply to all sites that deliver raw and derived PDB data, including PDBe.

## 3. PDBe focus areas

PDBe has identified five focus areas in which it either has traditionally been strong or wants to become strong in the near future, namely (i) advanced services, (ii) ligand annotation and analysis, (iii) integration and presentation of bio­molecular and other data, (iv) validation and (v) experimental data (presentation, validation and analysis). Some past activities and some ongoing and future developments in each of these areas are described below.

### 3.1. Advanced services

PDBe has established a reputation as a provider of advanced databases and services built upon the data available in the PDB and other resources, including the following.

(i) *PDBeFold* (Krissinel & Henrick, 2004*a* ▶,*b* ▶). This service can be used to compare a protein structure with all the protein structures in the PDB or against the domains covered by the SCOP structure-classification database (Andreeva *et al.*, 2008 ▶). The method is based on a subgraph-isomorphism-detection algorithm developed at PDBe and implemented in the program *SSM* (Krissinel & Henrick, 2004*a* ▶,*b* ▶). This algorithm is fast and allows structure comparison against the entire PDB in a matter of minutes. For every similar structure found, the output includes details of the matching secondary-structure elements and residues, and superimposed structures can be visualized.
(ii) *PDBePISA* (Krissinel & Henrick, 2007 ▶). This can be used for prediction and analysis of the probable quaternary structure of a crystal structure. It is also used in the annotation of structures in the PDB and replaces the older *PQS* service (Henrick & Thornton, 1998 ▶). All of the predicted quaternary structures can be downloaded and the server provides a detailed analysis of all interfaces. It is also possible to search similar interfaces in the entire PDB based only on the three-dimensional arrangement of the residues in an interface.
(iii) *PDBeMotif* (Golovin *et al.*, 2005 ▶; Golovin & Henrick, 2008 ▶, 2009 ▶). This service can be used for exploration of ligand-binding sites and small three-dimensional structural motifs across the entire PDB archive. *PDBeMotif* also integrates sequence-based annotation information using DAS technology (Dowell *et al.*, 2001 ▶) and allows users to compare this information with annotations based on the three-dimensional structure. It is possible to examine the ligand-binding characteristics of a single protein or of groups of proteins based on classifications such as EC (Fleischmann *et al.*, 2004 ▶), Pfam (Finn *et al.*, 2010 ▶), CATH (Greene *et al.*, 2007 ▶) and SCOP.

### 3.2. Ligands

The ligand-bound structures in the PDB offer unique insights into the molecular interactions of small molecules and biomacromolecules. In many instances ligands modulate the activity of the protein to which they bind, but sometimes their presence is an artefact of the experimental procedure (*e.g.* purification or crystallization). Nevertheless, even ‘accidental’ ligands can offer insight into how the natural substrate might interact with its biomacromolecular target. The process of identifying ‘interesting’ ligands will usually require manual annotation. PDBe has started a pilot project on the annotation of such ligand instances based on the simple rule that if a ligand and its environment are discussed in the primary publication of the PDB entry then it is likely to be ‘interesting’. The ligand is then classified as ‘biologically interesting’ or ‘experimentally interesting’ (*e.g.* a small molecule bound at a crystallographic special position) and this classification and supporting information are stored in a database for future use.

Presenting protein–ligand interaction data to the wider community presents another challenge, since understanding the three-dimensional nature of the interaction site is not straightforward. Approaches such as *LigPlot* (Wallace *et al.*, 1996 ▶) have been successful in making this information accessible as simplified two-dimensional diagrams with key details about the molecular interactions. The two-dimensional approach, while sophisticated, still has the limitation of not conveying the three-dimensional nature of the binding site. At PDBe we are working to combine customisable and linked two-dimensional and three-dimensional views. Together with annotation of ‘interesting ligands’ this will enhance the value of the PDB archive.

### 3.3. Integration

PDBe is part of the European Bioinformatics Institute (EBI), which is home to a number of core bioinformatics databases and services that hold data relevant to the bio­medical field (Brooksbank *et al.*, 2010 ▶). This puts PDBe in a unique position and enables it to enhance the annotation of biological data with insights from the macromolecular structure information available in the PDB and *vice versa*. This approach to data integration has been a mainstay of the bioinformatics field in the past decade (Chicure, 2002 ▶; Valencia, 2002 ▶). PDBe collaborates primarily with UniProt (UniProt Consortium, 2009 ▶) to integrate information from protein sequences and structures. This collaboration, which started in 2000, has resulted in a data resource called SIFTS (Structure Integration with Function, Taxonomy and Sequences; Velankar *et al.*, 2005 ▶). SIFTS is the authoritative source of up-to-date residue-level annotation of structures in the PDB with data available in UniProt, CATH (Greene *et al.*, 2007 ▶), SCOP (Andreeva *et al.*, 2008 ▶), GOA (Barrell *et al.*, 2009 ▶), InterPro (Hunter *et al.*, 2009 ▶) and Pfam. SIFTS itself is used by major resources such as Pfam, CATH, RCSB, DAS server providers (http://www.dasregistry.org/) and many research and service groups around the world. In the future, SIFTS might be extended to link PDB data to other resources such as ChEMBL (http://www.ebi.ac.uk/chembl/; protein–ligand interaction data), IntAct (Aranda *et al.*, 2010 ▶; macromolecular interaction data), Reactome (Matthews *et al.*, 2009 ▶; biological pathway data), ChEBI (De Matos *et al.*, 2010 ▶; ligand chemistry and function data) and EnsEMBL (Flicek *et al.*, 2010 ▶; SNPs and genetic variation data).

Although data-integration efforts have resulted in an infrastructure that allows the easy transfer of information and annotations between various bioinformatics resources, this in itself does not necessarily result in additional insight into the biological context, function or role of a given biomacro­molecule. Intelligent query and visualization mechanisms using modern tools and technologies are essential for making structure data relevant for the wider biomedical field. Efforts to develop such tools are under way at PDBe and some initial results are described in §[Sec sec4]4.

### 3.4. Validation

It is generally difficult for non-expert users of structural data to assess the reliability of the data and inferences based on a macromolecular structure (Kleywegt, 2009 ▶). To address this issue, PDBe will create a validation portal for biomacromolecular structure data. As a first step, the Uppsala Electron Density Server (EDS; Kleywegt *et al.*, 2004 ▶) will be integrated into the PDBe infrastructure. The results of the wwPDB validation pipeline will be combined with electron-density data and presented in an integrated validation viewer for X-­ray crystal structures. Similar functionality will be developed for structures determined by other experimental methods as the VTFs submit their recommendations to the wwPDB partners.

### 3.5. Experimental data

NMR contributes about 15% of the structures in the PDB archive. This technique presents several distinct challenges com­pared with X-ray crystallography, especially with regard to data complexity, consistent data storage and diversity of software. To encourage the community to tackle these issues before deposition, PDBe has been collaborating closely with the Collaborative Computational Project for NMR (CCPN) for the last decade (Fogh *et al.*, 2006 ▶; Vranken *et al.*, 2005 ▶). This work has resulted in a deposition system where NMR spectroscopists can upload a complete CCPN project to PDBe from which all information relevant to the PDB is extracted (Penkett *et al.*, 2010 ▶) and NMR data are forwarded to the BioMag­ResBank (BMRB; Ulrich *et al.*, 2008 ▶). These efforts on consistent data storage have also resulted in software to handle a large variety of NMR data formats (Vranken *et al.*, 2005 ▶) and are used in data-cleanup projects (Doreleijers *et al.*, 2005 ▶, 2009 ▶) and the CASD–NMR software-assessment competition (Rosato *et al.*, 2009 ▶). The large data archives created in this way have made it possible to carry out comprehensive data analyses (Vranken, 2007 ▶; Vranken & Rieping, 2009 ▶) and structure recalculations (Nederveen *et al.*, 2005 ▶; Nabuurs *et al.*, 2004 ▶) and have driven new developments in the field (De Simone *et al.*, 2009 ▶; Rieping & Vranken, 2010 ▶). PDBe continues to work with the NMR community to ensure that there is a public archive with structures that are supported by well defined experimental data. PDBe is also committed to delivering these data back to the community in forms that encourage further developments in the field of biomacromolecular NMR spectroscopy.

Since 2002, PDBe has worked closely with the EM community in establishing and developing the Electron Microscopy Data Bank (EMDB; Tagari *et al.*, 2002 ▶). Initially a purely European affair, the archive is now operated and developed jointly by the three EMDB partner sites PDBe, RCSB and BCM. A deposition and annotation system for EM data was developed at PDBe (Henrick *et al.*, 2003 ▶) and is now available at PDBe and RCSB. The EMDB partner sites are also working on integrating the three-dimensional structure data available in the PDB with EM volume data. The EMDB data can be accessed through the joint website at http://EMDataBank.org (Lawson *et al.*, 2011 ▶).

In addition to the presentation and analysis of NMR and EM data, the integration of EDS into the PDBe service infrastructure will deliver up-to-date electron-density maps and analyses based on the fit of models and data to the user community.

## 4. New developments at PDBe

**Figure 1:**
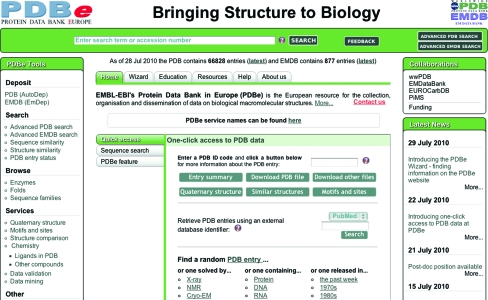
The new PDBe home page, where the top search bar provides a common interface for simple searches of both PDB and EMDB. The ‘Quick access’ panel allows users to perform the most common tasks such as finding detailed information about a particular PDB entry, searching the PDB by various database identifiers or searching the PDB based on a protein sequence. The menu on the left provides access to many of the PDBe search and browse tools.

PDBe has begun the process of addressing the needs of new communities of users who are not experts in structural biology, a process that is likely to take 5–10 years. In the summer of 2010, PDBe launched its redesigned website (http://pdbe.org; Velankar *et al.*, 2011 ▶; Fig. 1 ▶) which included a number of new features that may be considered first steps on the road to its becoming a useful resource for biomedicine. The home page of PDBe was completely redesigned, with the express aim of making it easier and more intuitive to locate information, resources and services, even for first-time non-expert users. This is achieved by offering menus that describe services and resources by their function rather than by an arcane name, by providing a single search box in the top bar on the home page which will search both PDB and EMDB simultaneously and by providing a number of quick-access tools to retrieve key data based on PDB ID code. The PDBe database can also be queried using database identifiers for various relevant resources such as PubMed, Pfam, SCOP, CATH, EC and InterPro. It is further possible to carry out *FASTA* (Lipman & Pearson, 1985 ▶) searches against all protein sequences in the PDB from the home page.

A tool to help new users of the website find the information that they are looking for is the PDBe Wizard (http://pdbe.org/wizard). The Wizard first tries to establish what the user is looking for and what information they already have. Based on this, it either provides the user with a box in which to enter some input (*e.g.* a PDB code, author name or UniProt ID) and start a search or it provides a link to a resource or service or a page with more information. At the stage where input is required, there are two helpful buttons. One, labelled ‘Shortcut’, provides hints on how to carry out the same search more quickly using PDBe services directly. The other, labelled ‘What results will I get?’, shows examples of what results can be expected and how they will be presented.

**Figure 2:**
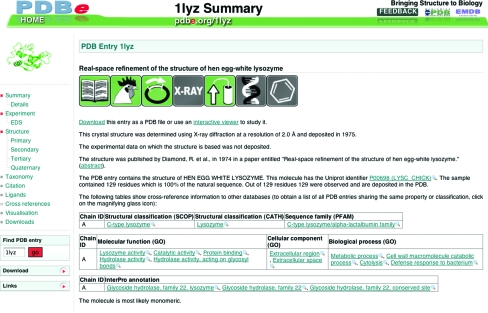
Example of an Atlas page, in this case for PDB entry 1lyz. The menu on the left-hand side enables navigation between different areas of information and provides links to other resources and downloadable files. The main panel on the right shows the summary information for the entry.

For every PDB entry there is set of ‘atlas’ pages that provide important information about the structure determination, the sequences of the biomacromolecules, the secondary structure of any proteins, the probable quaternary structure, literature references *etc*. The URLs for these pages are of the form http://pdbe.org/1cbs, where ‘1cbs’ is an example of a PDB ID code of interest. By default, a summary page will be presented that uses plain-English sentences to describe key aspects of the structure and the study, as well as some figures and tables with cross-references to other databases (Fig. 2 ▶).

**Figure 3:**
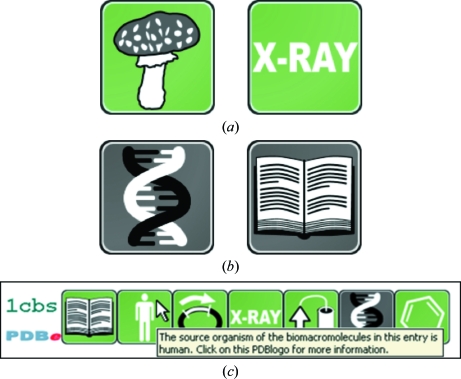
PDBlogos and PDBprints. (*a*) PDBlogos are stylized icons that convey important information about a PDB entry. For example, these two PDBlogos signify that the biomacromolecule in an entry derives from a fungus and that the structure was determined by X-ray crystallography, respectively. (*b*) By default, PDBlogos are shown on a green background (although this may be set to a different colour on external websites). However, sometimes the background will be grey: this signifies that either the feature symbolized by the PDBlogo is absent or that the underlying data are not available, not published or not deposited. For instance, these two PDBlogos show that an entry does not contain any nucleic acid molecules and that the structure has not (yet) been published, respectively. (*c*) A PDBprint for a PDB entry is a collection of PDBlogos displayed in a specific order, where each icon represents a well defined category of information (see text). This PDBprint shows immediately that PDB entry 1cbs is a published crystal structure of a heterologously expressed human protein in complex with a ligand for which the experimental diffraction data have beeen deposited. To help users familiarize themselves with the meaning of PDBlogos, tool tips are presented.

PDBe has introduced a new method to convey information about key aspects of a PDB entry using intuitive icons called PDBlogos (http://pdbe.org/pdbprints; Fig. 3 ▶). In order to make the interpretation of the information conveyed by PDBlogos easier and to provide consistent information when a number of PDB entries are compared, PDBe has also developed PDBprints. A PDBprint for a PDB entry is a collection of PDBlogos displayed in a specific order, where each icon represents a well defined category of information. In the first release of PDBprints (summer 2010) the following categories are included:

(i) Primary citation: has the PDB entry been published?
(ii) Taxonomy: what is the source organism of the bio­macromolecule(s) in the entry?
(iii) Sample-production technique: how was the sample of the biomacromolecule(s) obtained?
(iv) Structure-determination method: which experimental technique(s) was used to determine the structure and were the experimental data deposited?
(v) Protein content: does the entry contain any protein molecules?
(vi) Nucleic acid content: does the entry contain any nucleic acid molecules (DNA, RNA or a hybrid)?
(vii) Heterogen content: does the entry contain any ligands (such as inhibitors, cofactors, ions, metals *etc*.)?

Fig. 3 ▶(*c*) shows the PDBprint for PDB entry 1cbs, which shows that 1cbs is a published crystal structure of a heterologously expressed human protein in complex with a ligand for which the experimental diffraction data have been deposited.

In a first attempt to enable users to access the structure archive using familiar biological classifications and to analyse the selected structures in a number of ways, PDBe has introduced a database browser called *PDBeXplore*. At present, there are three browser modules based on the following classification systems:

(i) the enzyme-classification (EC) system as included in the IntEnz (De Matos *et al.*, 2010 ▶) database (http://pdbe.org/ec),
(ii) the sequence-based protein-family classification system (http://pdbe.org/pfam) and
(ii) the fold-based protein-family classification system CATH (http://pdbe.org/cath).

**Figure 4:**
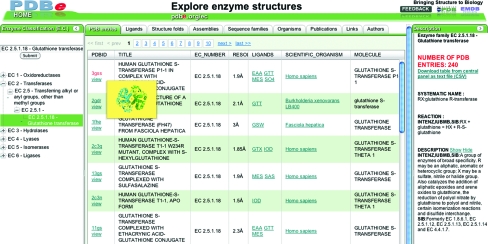
The PDBe enzyme-browser tool. The left-hand panel shows the EC classification as a tree and the right-hand panel gives detailed information on the selected class of enzyme. The central panel shows structure data relevant to the selected EC class organized as a number of tabs (*e.g.* ligands, quaternary structure, folds *etc*.).

**Figure 5:**
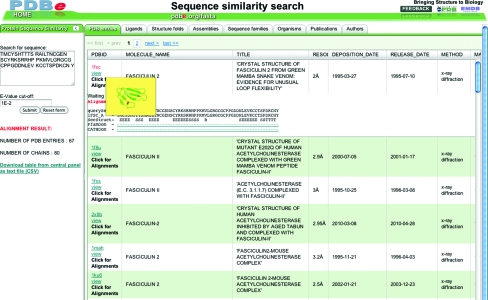
The PDBe browser for analysing the results of sequence searches against the PDB. The query sequence can be shown aligned with each of the target sequences, together with their secondary structure, Pfam and CATH domain annotations.

Fig. 4 ▶ shows a glimpse of the enzyme-browser functionality. The browser not only lists all the PDB entries that are relevant for a given query but also provides instant reports and analyses of the relevant structure data. These reports pertain to the distribution of probable quaternary structures, bound ligands, sequence-family data (based on Pfam), taxonomy and fold classifications. All of the information can be downloaded for further off-line use. PDBe offers similar browser functionality for sequence-based searches (http://pdbe.org/fasta). This tool offers a list of all PDB entries that include proteins that have a sequence similar to the query sequence and provides additional analyses and reports. The browser also shows the alignment of the query and target sequences with annotation about secondary-structure assignment and CATH and Pfam domains (Fig. 5 ▶).
